# Time Trends, Regional Variability and Seasonality Regarding the Incidence of Type 1 Diabetes Mellitus in Romanian Children Aged 0-14 Years, Between 1996 and 2015

**DOI:** 10.4274/jcrpe.5456

**Published:** 2018-05-18

**Authors:** Adrian Vlad, Viorel Serban, Anders Green, Sören Möller, Mihaela Vlad, Bogdan Timar, Alexandra Sima, on behalf of the ONROCAD Study Group

**Affiliations:** 1 Victor Babes University of Medicine and Pharmacy, Department of Diabetes and Metabolic Diseases, Timisoara, Romania; 2 Cristian Serban Medical Center of Evaluation and Rehabilitation for Children and Adolescents, Buzias, Romania; 3 University of Southern Denmark, Odense University Hospital, Department of Clinical Research, Odense Patient Data Exploratory Network (OPEN), Odense, Denmark; 4 Victor Babes University of Medicine and Pharmacy, Department of Endocrinology, Timisoara, Romania; 5 Victor Babes University of Medicine and Pharmacy, Department of Biostatistics and Medical Informatics, Timisoara, Romania

**Keywords:** Type 1 diabetes mellitus, Romania, children, incidence, seasonality

## Abstract

**Objective:**

The incidence of type 1 diabetes mellitus in children is highly variable in the world. The aim of our study was to: 1) analyze the evolution of the incidence of childhood type 1 diabetes in Romania between 1996 and 2015, and: 2) to search for differences amongst age groups, gender, geographic regions and month of diagnosis.

**Methods:**

Data on all new cases of type 1 diabetes, aged <15 years, obtained from two independent sources, were included in the study. The statistical methods included modeling of the incidence rates, adjusting for age, sex, calendar year, geographic region and seasonality.

**Results:**

The study group was composed of 5422 children, with overall completeness of ascertainment estimated at 93.7%. The incidence rate (per 100.000 person-years) rose continuously, from 4.7 [95% confidence interval (CI) 3.9-5.7] in 1996 to 11.0 (95% CI 9.9-12.2) in 2015, by a yearly rate of 5.1%, highest in the youngest and lowest in the oldest children. The mean incidence was significantly higher (p<0.0001) in Transylvania (7.9, 95% CI 7.6-8.3) than in Moldavia (6.5, 95% CI 6.2-6.9) and Muntenia (7.0, 95% CI 6.7-7.3), probably due to differences regarding ethnicity and lifestyle. The monthly incidence showed a sinusoidal pattern, peaking in January and being minimum in June.

**Conclusion:**

The incidence of type 1 diabetes mellitus in Romanian children increased continuously during the study period by a rate that, if maintained, would lead to its doubling every 14 years. Important differences were established between geographic regions and seasonality at diagnosis.

## What is already known on this topic?

The incidence of type 1 diabetes mellitus is highly variable in the world. Epidemiological data for the EURODIAB Study and the DIAMOND Project were collected in Bucharest, Romania’s capital city. Romania had a low incidence of the disease, but it increased continuously over a 10-year period (2002-2011) in children aged 0-17 years.

## What this study adds?

Our study found a steeper rise in the incidence of childhood type 1 diabetes mellitus than the one reported globally. Romania now belongs with the countries having a high incidence of the disease. There are significant differences between geographic regions and there is a seasonality regarding the diagnosis.

## Introduction

Knowledge about the epidemiology of type 1 diabetes mellitus (T1DM) has always been of interest among diabetologists. Epidemiological information is important not only for health care systems, but also for researchers, providing valuable information about the underlying mechanisms of this chronic disease. The currently accepted theory regarding the pathogenesis of T1DM states that, in genetically predisposed individuals, the intervention of some environmental factors triggers the activation of the immune system and leads to beta cell destruction and, consequently, to absolute insulin deficiency (1). However, the mechanisms are probably more complex, as there are cases of T1DM where insulin resistance probably plays an important role (2).

Most of the data regarding the incidence of T1DM concerns children, and they derive from two important multinational studies (3,4), as well as from national or regional reports. All these studies state that the incidence increases over time and is highly variable, depending on age, geographic region, and season. More specifically, T1DM is most common in children aged 10 to 14 years, has a probability of occurrence that increases with the distance from the Equator (suggesting the role of vitamin D deficiency), has its onset mainly in the cold season (underlining the role of viral infections), and increases by a mean annual rate of about 3%.

However, the knowledge regarding the secular trend of the incidence is far from being complete, since some data show continued increase (5,6), whereas others found that the increase has leveled off (7) or has a sinusoidal pattern (8).

Romania was part of the aforementioned multinational studies, but it provided data only from the capital, Bucharest, and its surroundings. In the last two decades, the Romanian Childhood Diabetes Registry was developed, and has provided information from all over the country. According to this source, Romania had a low incidence of the disease, with some regional differences (9), but the incidence has increased continuously over a 10-year period in children aged 0 to 17 years (10). The most recent data suggests that Romania may now be included in the group of countries that have a high incidence of pediatric T1DM (11).

The aim of this paper is to characterize the evolution of the incidence of T1DM in children aged 0 to 14 years during the 20 years that the Romanian Childhood Diabetes Registry has existed, and to establish the possible differences between various age groups and the two genders, as well as to reveal some regional and seasonal particularities.

## Methods

This retrospective study was conducted on Romanian boys and girls under the age of 15, diagnosed with T1DM between 1996 and 2015. The study was approved by the Ethical Committee of “Victor Babes” University of Medicine and Pharmacy Timisoara (approval number: 43/11.05.2016). Due to the fact that it was a retrospective epidemiological study, informed consent was not requested.

The cases were collected from two independent sources. The primary source was the Romanian Childhood Diabetes Registry. This registry was started in 1996 by ONROCAD (Romanian acronym for the “Romanian National Organization for the Protection of Children and Adolescents with Diabetes”), and revised on a yearly basis, relying on the reports of the physicians who were managing these cases. Romania is divided into 41 administrative districts. Medical care for diabetic children is provided in a centralized manner. According to the rules imposed by the National Health Insurance Company, insulin and glucose strips are reimbursed only if prescribed by a limited number of physicians, up to four in each district (this person could be a pediatrician, a diabetologist or an endocrinologist). Consequently, the primary source of this study was based on the yearly reports of about 70 health care professionals, which included children with T1DM who constituted the majority of the cases compared to other types of diabetes. The records of the “Cristian Serban” Medical Center in Buzias, a public health care center specialized in the diagnosis, evaluation, education and treatment of children and young people with diabetes mellitus from all over the country, considered as a European Reference Center for the management of pediatric diabetes, constituted our second source (12).

The completeness of ascertainment of the cases was calculated by the capture-recapture method (13), using the number of patients diagnosed by each of these two sources to estimate the number of missing patients and the total number of children.

The diagnosis of T1DM was established by the lead physician for each case, based on internationally accepted guidelines (14). The date of onset of the disease was considered to be the date of the first insulin injection. Cases that were younger than six months old at diagnosis were excluded, since the probability of developing T1DM before this age is very low.

### Statistical Analysis

The demographic data was retrieved from the National Institute of Statistics (15). These data were derived from the censuses performed in 1992, 2002, and 2011, also using estimates for the other years. The data were collected for each of the 41 administrative districts, for both genders, and for three standard age groups: 0-4 years, 5-9 years, and 10-14 years.

The incidence rates were expressed as new cases per 100.000 person-years at risk, with approximate 95% Poisson confidence intervals (CIs). Poisson regression was used to model the incidence rates, adjusting for age groups, sex, calendar year and geographical region, taking the respective background population into account as exposure.

The analysis of seasonality at diagnosis was based on all cases aggregated over sexes and all years, with stratification by age at diagnosis (0-4 years, 5-9 years, 10-14 years), as well as all ages combined. Using methodology described by Edwards (16), the analysis included a general test for seasonal variation [Total, 11 degrees of freedom (df)], a test for sinusoidal variation (Cyc. trend), as well as a test for seasonal variation unexplained by lack of fit of the sinusoidal model (Residual, 9 df).

## Results

### Completeness of Ascertainment

After the exclusion of other types of diabetes mellitus, the primary source (the Romanian Childhood Diabetes Registry) identified 5248 cases, whereas the secondary source (the medical records from the “Cristian Serban” Medical Center in Buzias) provided 1878 cases. Among the total 5422 cases ascertained, 1704 were captured independently by both sources. Based on these data, the total number of patients captured by the two sources was 5422, the number of missed cases was estimated to be 362, and the probable total number of patients was 5784.

The overall completeness of ascertainment was 93.7% (95% CI 93.1-94.4)  and constant during the study period (data not shown). The value of the completeness of ascertainment for the primary source was 90.7% (95% CI 89.4-92.1), and for the secondary source 32.5% (95% CI 31.2-33.7).

### Trends in the Incidence of Type 1 Diabetes Mellitus Between 1996 and 2015

The yearly incidence rate (per 100.000 person-years) showed a continuous increase during the study period, from 4.7 (95% CI 3.9-5.7) in 1996 to 11.0 (95% CI 9.9-12.2) in 2015 (Table 1), corresponding to an annual mean increase of 5.1% (95% CI 4.6-5.6). This phenomenon was due to the absolute increase in the number of new cases of T1DM, as well as to a decrease in the target population. The rise of encountered in all age groups, reaching peak values in the youngest children [annual rate 7.6% (95% CI 6.3-8.9)], and the lowest values in children aged 10-14 years [annual rate 3.9% (95% CI 3.1-5.8)]. The increasing trend affected both boys and girls, without significant differences between them, and it was present in all the geographic regions of the country.

The mean incidence of the disease in the study period was 7.2 (95% CI 7.0-7.4). It was not significantly (p=0.53) higher in boys (7.3, 95% CI 7.0-7.5) than in girls (7.1, 95% CI 6.9-7.4). The differences between the age groups were statistically significant (p<0.0001). The highest incidence was found in the age group 10-14 years (9.1, 95% CI 8.7-9.4), followed by the age groups 5-9 years (7.6, 95% CI 7.3-8.0), and 0-4 years (4.5, 95% CI 4.2-4.7). The mean age at diagnosis was similar in girls and in boys (p=0.81).

### Regional Differences in the Incidence of Type 1 Diabetes Mellitus

**Figure 1:**
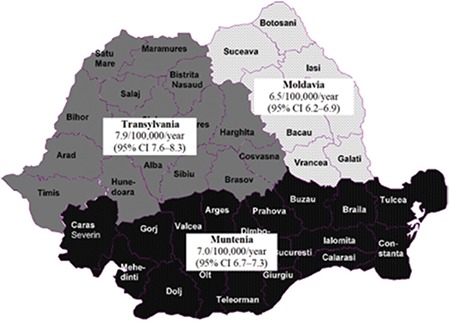
Regional differences in the incidence of type 1 diabetes mellitus in Romanian children aged 0-14 years, in the period 1996-2015 CI: confidence interval

There were striking differences between geographic regions regarding the incidence of T1DM (Figure 1). The mean incidence during the study was significantly higher (p<0.0001) in Transylvania (7.9, 95% CI 7.6-8.3), in comparison to Moldavia (6.5, 95% CI 6.2-6.9) and Muntenia (7.0, 95% CI 6.7-7.3).

### Seasonal Variation in the Incidence of Type 1 Diabetes Mellitus

**Figure 2:**
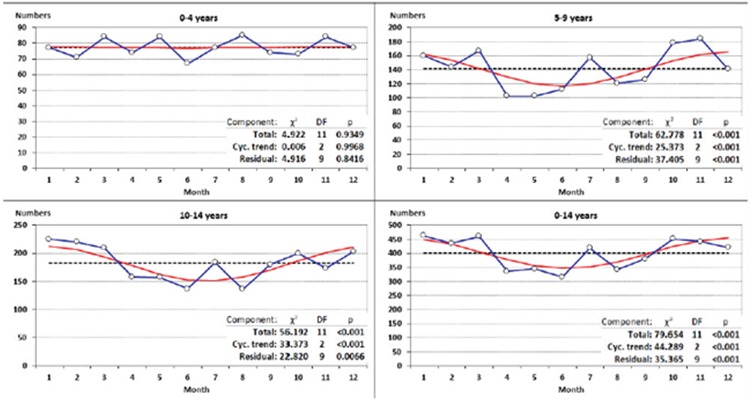
Seasonality of the incidence of type 1 diabetes mellitus in Romanian children aged 0-14 years (1996-2015). Blue lines with marks are observations, read lines are best fitted sinusoidal curves, dotted black lines are monthly average (ignoring differing days in months) For each age group χ^2^-values with corresponding degrees of freedom and p values are presented as total (test of heterogeneity by month), Cyc. trend (significance of accepting a sinusoidal model), and residual (test of variation unexplained by the sinusoidal model)

The incidence of T1DM differed by months in a pattern compatible with a sinusoidal variation (Figure 2). The incidence was higher during winter, with the maximum value registered in January, and lower values during summer, with the minimum value encountered in June. The pattern of seasonal variation was significant (p<0.001) for all age groups except for the youngest (0-4 years), however with statistically significant evidence of residual variability.

## Discussion

The epidemiology of T1DM in Romanian children has been described previously. The first papers included only the capital, Bucharest (17), this region being part of the two important international epidemiological studies, EURODIAB (4) and DIAMOND (3). Later, data covering the whole country also became available (9,10). The present update is justified by the enlarged study material, now covering a full 20-years period from 1996, by the need to confirm the previously published age, gender and calendar time trends, and by the necessity to address the geographical distribution within Romania and the seasonality at diagnosis.

### Completeness of Ascertainment

The incident cases were retrieved from two sources that diagnose and report new cases of childhood T1DM independently from each other. The primary source was the Romanian Childhood Diabetes Registry, in which records of children with diabetes are included, based on the evidence kept and forwarded by local diabetologists or endocrinologists. The secondary source was represented by the data files of patients admitted to the “Cristian Serban” Medical Center in Buzias, a public hospital where children with diabetes mellitus are admitted if referred by a physician or by direct registration via the diabetes association or website. The overall completeness of ascertainment was high (93.7%), and it was constant over the study period. The primary source had, by itself, a high completeness of ascertainment (90.7%). This could be explained by the fact that the medical care of children with diabetes mellitus is provided by a health system that can be considered centralized, as each of the 41 districts includes only few physicians (up to four) who are in charge of these cases. The relatively low number of physicians facilitates the achievement of almost complete data for the Romanian Childhood Diabetes Registry. The low completeness of ascertainment of the “Cristian Serban” Medical Center in Buzias may be explained by the fact that only a small percentage (about 30%) of all the children with diabetes from Romania benefited from evaluation, education and treatment within this hospital.

### Trends in the Incidence of Type 1 Diabetes Mellitus Between 1996 and 2015

During the 20 years of follow-up, the incidence of T1DM in children increased constantly, with an annual rate of 5.1%, which is higher than the one reported by the EURODIAB study and the DIAMOND project (3,4). Due to the high completeness of ascertainment one can consider this information accurate and exclude bias due to under-reporting. In the past, Romania was included in the group of countries with a low incidence of T1DM in children (9), but, as shown by the present data, the country presently has a high incidence of this disease.

Such a rapid increase in incidence rates usually characterizes countries with lower overall incidence (18), while in those where the disease is more prevalent, the incidence rate increases more slowly or even levels off (6,7). However, in Romania, the increase continued to be steep even when the incidence became high, and, if this rate is maintained, one can expect a doubling of the incidence rate every 14 years. Even without valid information, one can speculate that, in the past, the incidence rate of T1DM was approximately constant, probably at a low value, and at a certain moment it started to increase (19). That specific moment was probably shortly after 1990, when the political changes in Romania led to important changes in lifestyle, represented by a higher mobility of the population and by the increased use of industrially processed food. These might have led to a greater exposure to infectious agents and to chemical substances found in food, putative triggers for the autoimmune destruction of the beta cells. It is very probable that new and different environmental factors contributed to the marked increase in the incidence of T1DM, as the study period is too short for the occurrence of genetic changes that could explain this rise in incidence. Enhanced economic development has been postulated as the reason for rapid increases in the incidence of T1DM by other authors (20).

It is known that Hungary (21) and Germany (22) have higher incidences of childhood T1DM compared to Romania. One can assume that the Hungarian and German ethnicities in Romania (two of the most important ethnic minorities), who have a similar genetic background to the population from the aforementioned countries, might have higher incidences of T1DM and that an increase in the percentage of those ethnicities might explain the rise in the incidence of T1DM. However, this demographic phenomenon was not shown by national censuses (15), which demonstrated, in fact, a decrease in the percentage of these populations, as a consequence of their emigration to Hungary and Germany, respectively.

Another possible explanation for this steep increase is the shift to a younger age at diagnosis. It is hypothesized that T1DM occurs at a younger age, due to an earlier effect of the environmental triggers, and that the overall incidence of the disease is not changed. This idea is supported by several trials that have analyzed the evolution of the incidence of T1DM in children, as well as in adults (23), and was suggested by previous work carried out by our group, in which the analysis performed over a 10-year period reported an increase in the incidence of T1DM in the age groups 0-4, 5-9 and 10-14, and a decrease in the age group 15-17 (10). In the present study, the more rapid increase in the incidence of T1DM in the youngest children may be evidence to support the theory of the shift towards a younger age at diagnosis.

The highest incidence of T1DM was registered in the age group 10-14. This is the age when most of the children reach puberty. The insulin resistance induced by the release of sex hormones may play an important role in the onset of T1DM (2). Boys had a higher incidence of T1DM in most years and in all age subgroups. This is usually the case for populations with high incidence of the disease (24), without a clear explanation for this phenomenon. The mean age at diagnosis was significantly lower in girls as compared to boys. It is known that girls usually reach puberty at a younger age in comparison to boys, and this might be an explanation for the earlier onset. Our data are in concordance with reports of other authors regarding this (25).

### Regional Differences in the Incidence of Type 1 Diabetes Mellitus

Romania is composed of three geographic regions (Transylvania, Moldavia and Muntenia), each of them including several administrative districts. Transylvania was, in the past, part of the Austro-Hungarian Empire. Nowadays, its population is more heterogeneous, as compared to Moldavia and Muntenia, the Hungarian and German ethnic minorities being more numerous here than in the other regions.

Our study has revealed significant differences between these three regions. The highest mean incidence was encountered in Transylvania, and this was significantly higher compared to Moldavia and Muntenia, respectively. In a previous paper published by our group (9), an analysis performed on these three geographic regions, between 1992-1995, showed similar differences, though the mean rates of incidence were much lower. The main explanation for this finding might be the ethnic heterogeneity of Transylvania, knowing that children from Hungary and Germany (who have a genetic background similar to the aforementioned minorities from our country) have higher rates of T1DM in comparison to Romania (21,22). However, we cannot test this hypothesis further, since neither the primary nor the secondary source contains information about the ethnicity of the patients. Another possible explanation could be the fact that Transylvania is a region with a higher standard of living, compared to the other two regions and, consequently, one may speculate that its population has a higher mobility (more frequent trips in different regions of the world), and adopted more modern eating habits, based on industrial processed food. These differences could facilitate the interaction of some environmental factors (infections, food antigens) in a genetically predisposed population and trigger the autoimmune destruction of pancreatic beta cells.

### Seasonal Variation in the Incidence of Type 1 Diabetes Mellitus

The seasonal variability of T1DM underlines the role of infectious triggers and of the deficit of sun exposure in the pathogenesis of the disease (26,27). The putative infectious agents, notably viruses, are more common and long-lived in winter; consequently, given their involvement in the pathogenesis, the number of new cases should be higher in winter. In addition, the reduced number of sunny days in the winter induces a deficit in the subcutaneous synthesis of vitamin D, known to have an important role in immune modulation (28). However, due to the fact that the time elapsed between the intervention of the trigger and the onset of hyperglycemia is highly variable, the seasonal variability is not always obvious.

Our study has revealed significant seasonal variation of the incidence of T1DM, the highest values being recorded during winter (maximum incidence in January), and the lowest during summer (minimum incidence in June). This pattern was seen in children belonging to the age groups 5-9 and 10-14, but not for the youngest ones. The quite constant occurrence of the disease between ages 0-4 could be explained by the decreased exposure to infections, due to country’s specifics: children are raised by their parents in the first two years of life (this is the length of the parental leave), by their grandparents in the following year. Admission to communities, where infections are more prevalent, usually takes place after the age of three years.

Similar data were reported by the EURODIAB investigators (27) for the majority of the 23 member centers, except for two amongst which Romania’s capital, Bucharest. Since our study periods overlap substantially (1989-2008 in EURODIAB and 1996-2015 in our study) and the meteorological conditions do not differ significantly in Bucharest from the rest of the country, the different results could be explained by a lower prevalence of vitamin D deficiency in Bucharest, possibly due to better preventive measures.

### Study Limitations

Our study has several limitations and strengths. The main limitations are represented by the lack of information regarding the ethnicity of the newly diagnosed patients, a finding that could have facilitated the interpretation of the regional differences in the incidence. The absence of data about the dietary habits in early life and of the population from different geographic regions, as well as the lack of evidence about the evolution of the incidence of diabetes mellitus in adolescents and young adults, a finding that could have supported the theory of the shift towards a younger age at diagnosis, can be listed as other limitations. The strengths of our research consist in the existence of accurate data, provided by two high quality sources that cover the entire territory of the country over a long period.

## Conclusion

We have analyzed the trends in the evolution of the incidence of T1DM in Romanian children aged 0-14 years, over a 20-year span (1996-2015), taking into consideration some particularities regarding age group, gender, geographic region, and seasonality. We found a steeper rise in the incidence than the one reported globally, for both genders and all age groups and, if this trend were to be maintained, it would lead to a doubling of the incidence every 14 years. Romania now belongs with the group of countries with a high incidence of childhood T1DM. There are significant differences between the geographic regions and there is a seasonality regarding the diagnosis of T1DM that concerns all age groups, except for the very youngest. The shortcomings of the research could be overcome by continuation of follow-up and the addition of new information about adult patients, ethnicity of the subjects and lifestyle with particular emphasis on changes in eating habits.

## Figures and Tables

**Table 1:**
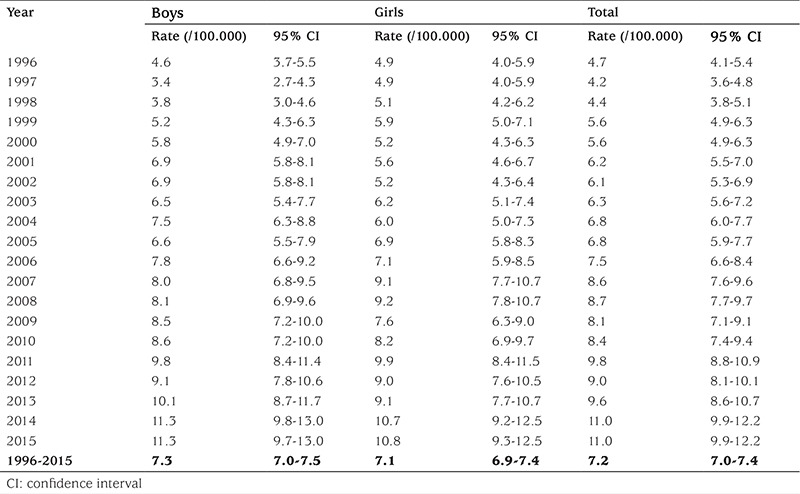
Crude incidence rates for type 1 diabetes mellitus in Romanian children aged 0-14 years, between 1996 and 2015
